# Nanoparticles and Inflammation

**DOI:** 10.1100/tsw.2011.106

**Published:** 2011-06-09

**Authors:** Ross Stevenson, Axel J. Hueber, Alan Hutton, Iain B. McInnes, Duncan Graham

**Affiliations:** ^1^ Centre for Molecular Nanometrology, Department of Pure and Applied Chemistry, WestCHEM, University of Strathclyde, Glasgow, UK; ^2^ Centre for Rheumatic Diseases, University of Glasgow, Glasgow, UK

**Keywords:** nanoparticles, inflammation, in vivo imaging

## Abstract

The development of nanoscale molecular probes capable of diagnosis, characterization, and clinical treatment of disease is leading to a new generation of imaging technologies. Such probes are particularly relevant to inflammation, where the detection of subclinical, early disease states could facilitate speedier detection that could yield enhanced, tailored therapies. Nanoparticles offer robust platforms capable of sensitive detection, and early research has indicated their suitability for the detection of vascular activation and cellular recruitment at subclinical levels. This suggests that nanoparticle techniques may provide excellent biomarkers for the diagnosis and progression of inflammatory diseases with magnetic resonance imaging (MRI), fluorescent quantum dots (QDs), and surface enhanced Raman scattering (SERS) probes being just some of the new methodologies employed. Development of these techniques could lead to a range of sensitive probes capable of ultrasensitive, localized detection of inflammation. This article will discuss the merits of each approach, with a general overview to their applicability in inflammatory diseases.

## INTRODUCTION

Inflammation is the net result of a cascade of highly regulated events propagated upon stimulation, and is the major process through which the body repairs tissue damage and defends itself against foreign materials. Acute inflammation is typically caused by an external chemical, mechanical, or pathogenic influence; has a relatively short duration (hours to days); and is a necessary protection tool that removes foreign bodies and damaged tissue, preventing further damage. Aberrant/chronic inflammation requires no external stimulus and can cause a range of painful and debilitating symptoms. Further, such uncontrolled inflammation is often indicative of a more serious, underlying cause whose analysis may be used as a diagnostic marker for a number of conditions, including autoimmune, infectious, neurological, cardiovascular, and metastatic diseases. To achieve the very best therapy for each, it is critical to detect inflammation at an early, subclinical stage. In a subclinical stage of disease, no clear findings, such as arthritis of a joint or inflammation of the skin, are visible. However, histological changes will have occurred with aberrant cell distribution in targeted tissues. Histological analysis of chronic inflammation often indicates localized populations of macrophages and lymphocytes, as well as compromised blood vessels, fibrosis, and tissue necrosis. Critically, early diagnosis can give disease-specific information, key to making the best decisions relating to the necessity and intensity of therapy, and to subsequent prediction of outcomes.

Macrophage cells are key members of the mononuclear phagocyte system (MPS) and play a number of crucial roles in inflammation, including phagocytosis, antigen presentation, and the production of various cytokines and immune regulators[1]. The MPS contains monocytes, which circulate the blood and upon maturation often become macrophages, and resident tissue macrophages that can take many forms. Resident macrophages are found in all organs and connective tissues, and they have a major function in the primary immune response of tissues. Their activation secretes chemokines that adhere to circulating monocytes, trafficking MPS cells to the inflammation site and generating an immune response. The ability to visualize the migration of MPS cells would be advantageous both therapeutically and diagnostically, as alterations in macrophage clearance contribute to many common disorders, including artherosclerosis, autoimmunity, and major infections[2]. Since macrophages are phagocytotic, their labeling is a relatively straightforward task, requiring no transfection agents and, as discussed at length later, this has been exploited in a number of nanoparticle strategies[3]. The cytokines produced by macrophages not only sequester other immune cells to the tissue, they can also propagate an autoimmune response and inflammation. Cytokines maintain part of a complex regulatory network, and disruption of the balance between pro- and anti-inflammatory cytokines has been implicated in the pathogenesis of a number of inflammatory disorders[4]. This can be illustrated with tumor necrosis factor-$\alpha$ (TNF-$\alpha$), a cytokine implicated to play a key role in rheumatoid arthritis (RA). Targeting TNF-$\alpha$ as a central product of macrophages has been demonstrated as a powerful approach to treat RA, with many drug treatments utilizing antibodies or receptor fusion proteins to TNF-$\alpha$.

Advances in nanoparticle imaging have provided probe approaches capable of subclinical levels of detection of cellular recruitment and vascular activation or leakage[5]. It has been suggested that permeable vascular walls can aid nanoparticle localization at the site of inflammation and this has been exploited by the use of untargeted nanoparticles whose recruitment by macrophages has allowed the activated cells to deliver their probe payload to the site of inflammation. Alternatively, it is possible to accumulate nanoparticles at the inflammation locale by the use of surface-modified probes that have been functionalized to target specific analytes present at the active site. Both targeted and untargeted approaches have been applied to a range of techniques, including MRI, fluorescence, and Raman spectroscopy, for the detection of inflammation. Other techniques commonly used, but incapable of diagnosing the subclinical levels necessary for improved therapies, include computed tomography (CT), positron emission tomography (PET), and single photon emission computed tomography (SPECT)[6]. This review will discuss a number of the nanoparticle approaches applied to the detection of inflammatory disorders.

## NANOPARTICLES

Nanoparticles are typically characterized as materials that have at least one dimension in the scale of 0.1–100 nm. Synthetically produced from a range of materials, including metals, polymers, silica, phospholipid bilayers, liposomes, and inorganic dyes, nanoparticle fabrication is generally characterized as either a bottom up or top down preparation. Bottom up refers to the aggregation of atoms to form a stable population of molecular clusters, whereas the top down preparation relies on the destruction of a larger macromaterial into nano-sized units. For a comprehensive review of both approaches, see the report by Euliss et al.[7].

Although generally considered inert, it is necessary to appreciate the potentially deleterious effects of nanoparticles to human health. The application of nanoparticles to humans for diagnostic applications is an area of research still in its infancy[8] and it is noteworthy that adverse health effects have been observed from $TiO_2$ nanoparticles. Although not used for diagnostic purposes, $TiO_2$ nanoparticles that are commonly used as whitening or brightening agents in materials, such as sunscreen, toothpaste, paint, medicine, and foods, have been implicated in a number of undesirable genotoxic and cytotoxic effects in cultured human cells, as well as pulmonary fibrosis and tumors in animal models[9,10]. The observed effects in these studies may not be size specific, but more likely substrate specific, and it is noteworthy that a large body of evidence regarding the toxicity of gold nanoparticles, the most common substrate for Raman analysis, show no such undesirable effects, although contradictory reports also exist[11].

Nanoparticles have widely varying physical and chemical properties that can affect their *in vivo* properties. Scale, surface area to mass ratio, and surface charge can all have consequences on the interaction of nanoparticles to a host organism. Nanoparticle size has been implicated to have a number of important biological affects, including interaction with the reticuloendothelial system (RES), adjuvant properties, and ease of phagocytosis. For instance, early research has demonstrated that particles around 50 nm or greater are more readily taken up by macrophage cells than smaller equivalents[12]. Similarly, surface charge has been shown to affect toxicity, clearance, immune cell stimulation, and the binding of plasma proteins to the nanoparticles[13]. Metz et al. demonstrated that negatively charged nanoparticles are more easily recruited by macrophages than neutral species[12]. Further, in most applications, it is possible to enhance the bioavailability and biodistribution of nanoparticles in blood by the introduction of an inert coating group. Polyethylene glycol (PEG) is classic example and has been used for a number of years to improve the pharmacokinetic properties of drugs. PEGylation is believed to work by preventing the adsorption of plasma proteins, markers for the sequestration of particulates, to the surface and inhibiting nanoparticle removal from blood, with increases of nanoparticle half-life *in vivo* readily observed[14].

Although untargeted or PEGylated nanoparticles offer significant imaging opportunities, it is possible in most cases to enhance targeting abilities by attaching antibodies, antigens, or some other form of affinity reagent. Nanoparticles are generally designed to have a surface (or surface groups) amenable to modification. Careful choice of functionalizing ligand can allow the preparation of probes capable of acting as nanosensors, delivery vehicles, guides for surgical procedures, or as probes capable of sensing molecular responses. Nanomedicine, the application of nanoparticles for medicinal benefit, is a blossoming area of research and many reviews are available[2,15]. This review will focus largely on the application of nanosensors for the early detection of inflammation. At the most basic level, a nanosensor is a nanoparticle that can be observed in a sensitive manner. Example pairings include superparamagnetic agents as detected by magnetic resonance imaging (MRI), quantum dot (QD) detection with fluorescent microscopy, and coinage metal nanoparticles used in surface enhanced Raman scattering (SERS) analysis. It is possible to tune each of the aforementioned techniques for use with inflammation by modification of the surface with targeting groups.

## Nanoparticles for Inflammatory Drug Delivery

Although providing little to no detection signal, it is worthwhile commenting on some of the advances of using nanoparticles to deliver drugs for inflammation. RA is a chronic and progressive autoimmune disorder characterized by inflammation and destruction of joints. Therapeutic drugs are available; however, their benefits are limited by the adverse side effects observed from long-term use at high doses. A better, yet less practical, approach is to administer the drugs directly to the site of inflammation using interarticular injections that require skillful applications at a number of sites, making it an expensive therapy with regard to both time and cost. The most desirable approach would be the development of vectors, such as nanoparticles, capable of targeting and delivering their payload directly to the site of inflammation. Nanoparticles such as liposome-encapsulated drugs have been shown to be potential candidates, and have been used to encapsulate and deliver clodronate and glucocorticoid drugs to target arthritis in an animal model[16]. In another arthritis model, Richards et al. showed that i.v. administered clodronate liposomes could suppress the onset of disease by targeting macrophages[17]. An alternative approach encapsulated the glucocorticoid prednisolone in PEG-coated liposomes and administered the nanoparticles to adjuvant-induced arthritic rats. Analysis suggested that the nanoparticle approach was tenfold more effective than the "free" control, with disappearance of symptoms after 2 days and complete remission after 6 days. Similar to the other approaches discussed in this review, coating liposomes with PEG polymers enhances stability *in vivo* and has led to a range of long-circulating drug carriers known as sterically stabilized liposomes[18].

## ADVANCES IN NANOSENSORS

### Magnetofluorescent Nanoparticles

Magnetic nanoparticles (2 nm to 1 µm) and the use of MRI have been at the forefront of the development of nanoparticles for *in vivo* imaging. MRI is a noninvasive imaging modality capable of screening soft tissues with high spatial resolution and excellent contrast. It has the ability to differentiate chemical composition, water content, physical state, and molecular motion or diffusion. Unlike earlier imaging approaches, ionizing radiation is not necessary, thus MRI lends itself to safe temporal analysis. MRI contrast agents are generally para- or superparamagnetic materials capable of altering the relaxation times of water molecules, and giving rise to an increase or decrease in signal intensity depending on the "weighting" of the image. The most common contrast agents are chelated gadolinium (Gd) (III) complexes and iron oxide nanoparticles (IONs) encased in a biocompatible, inert dextran or polymer shell[19].

Chelates of Gd, consisting of a targeting agent on one or more Gd chelate, are the more commonly used imaging agents in MRI (Fig. 1). Their small size allows them to cross the endothelial barrier into interstitial spaces, offering unique imaging regions over larger nanoparticles and other imaging techniques; however, larger Gd payloads are generally required for modest gains[19]. It is essential that chelates are thermodynamically and kinetically inert and stable due to the reported discovery of a rare Gd-related toxicity known as nephrogenic systemic fibrosis[20]. The introduction of nanoparticle carriers, including micelles and liposomes, are thought to enhance Gd delivery, thus facilitating lower dosages. Analysis carried out by Sipkins et al., in their detection of tumor angiogenesis, used Gd probes targeted to α-V β-3 and showed that it is possible to load substantial payloads with the aid of liposomes[21]. α-V β-3 is an integrin expressed by platelets that is up-regulated in malignant melanomas.

Gd chelates have also been found useful in the study of atherosclerosis. The conventional method of atherosclerosis diagnosis and assessment is coronary angiography, an invasive method that provides only limited information with regard to the levels of inflammation[22]. Further, the most lethal acute manifestations of coronary artery disease (unstable angina, acute myocardial infarction) are most often not caused by atherosclerotic plaque itself, but rather the formation of a thrombus (blood clot) as an intrinsic result of plaque rupture, as depicted in Fig. 2[23]. Such thrombi contain significant quantities of fibrin, making it a useful molecular imaging target[19]. The successful targeting and subsequent visualization of thrombi using Gd agents *in vivo* was accomplished by Botnar et al.[24]. The authors utilized a rabbit model, and induced the rupture of atherosclerotic plaque and subsequent thrombosis using Russell's viper venom. A thrombin-targeting Gd chelate was formed using a commercially available peptide chain and Gd-diethylenetriaminepentaacetate (DTPA). This allowed the targeting and MRI imaging of both acute and subacute thrombosis, significant in the early clinical detection of fibrin-rich thrombi, potentially allowing more rapid and effective diagnosis and treatment.

The alternative contrast agents commonly used in MRI imaging are IONs, whose magnetic and biological properties are directly related to the particle size (2–4000 nm), shape, polydispersity, surface properties, and charge[25]. In the field of molecular imaging, it is primarily the smallest size bracket that is of interest: monocrystalline IONs (MIONs), consisting of a single 2- to 3-nm iron oxide crystal[19]. Due to their small size, such nanoparticles can avoid clearance by the RES, resulting in a preferable, high blood residence time. MIONs are commercially available with a variety of surface chemistries, allowing the covalent attachment of targeting ligands and, with respect to inflammation imaging, IONs have been used in two approaches. The first approach uses passive untargeted nanoparticles (as described earlier), relying on vascular leakage or uptake by phagocytotic cells to deliver the probes to the site of inflammation.

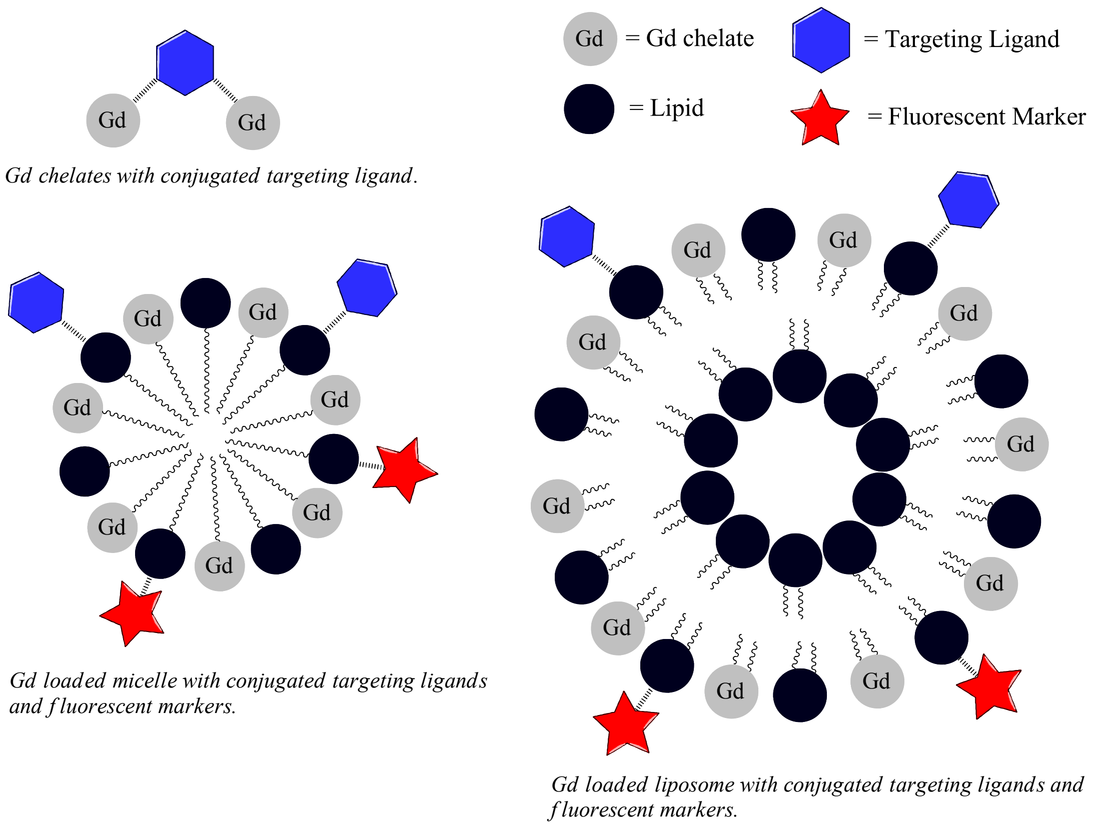

**FIGURE 1** Schematic illustration of three Gd-based agents[19].

The second relies on the abilities of functionalized MIONs to immune cells to actively target the site of inflammation. An example of the latter can be demonstrated by McAteer et al.[26], who functionalized tosyl-activated IONs with monoclonal antibodies directed against vascular endothelial adhesion molecules, including P-selectin and VCam-1, that are involved in leukocyte recruitment during early vascular inflammation. Using similar approaches, the same authors also applied MION probes to a number of clinically important vascular pathologies, including atherosclerosis, acute vascular inflammation, and ischemic stroke[26].

IONs have also found use in studies concerned with the imaging of atherosclerotic plaques. As discussed, macrophage-rich plaques are prone to rupture, which is likely to lead to myocardial infarction, and the ability to identify plaques that are at risk of rupturing could allow treatment or at least provide forewarning of acute cardiovascular events[27,28]. The ability to target, image, and assess the levels of these unstable macrophage-rich plaques accurately would be highly beneficial and MIONs have shown promise in allowing such a method. In a study by Ruehm et al., MIONs were shown to be phagocytosed effectively by atherosclerotic plaque macrophages, resulting in susceptibility changes detectable by MRI[29]. A similar approach by Korosoglou et al. demonstrated that MIONs could be utilized as positive contrast agents for inversion recovery with ON-resonant water-suppression MRI to enhance imaging of macrophage-rich plaques[28]. Morishige et al. also developed a tool capable of detecting macrophage burden in atheroma and detection of inflamed plaques[30]. Furthermore, they also found that the magnitude of signal enhancement is notably related to the macrophage concentration within the plaques. The implication of this is that their method constitutes a noninvasive means of determining the presence and progress of atherosclerosis and inflammation levels. The illustrated studies noted that similarities between rabbit and human atherosclerotic plaque formation provided good controls, and suggested the methods developed could be appropriate for the treatment and monitoring of atherosclerosis in humans in

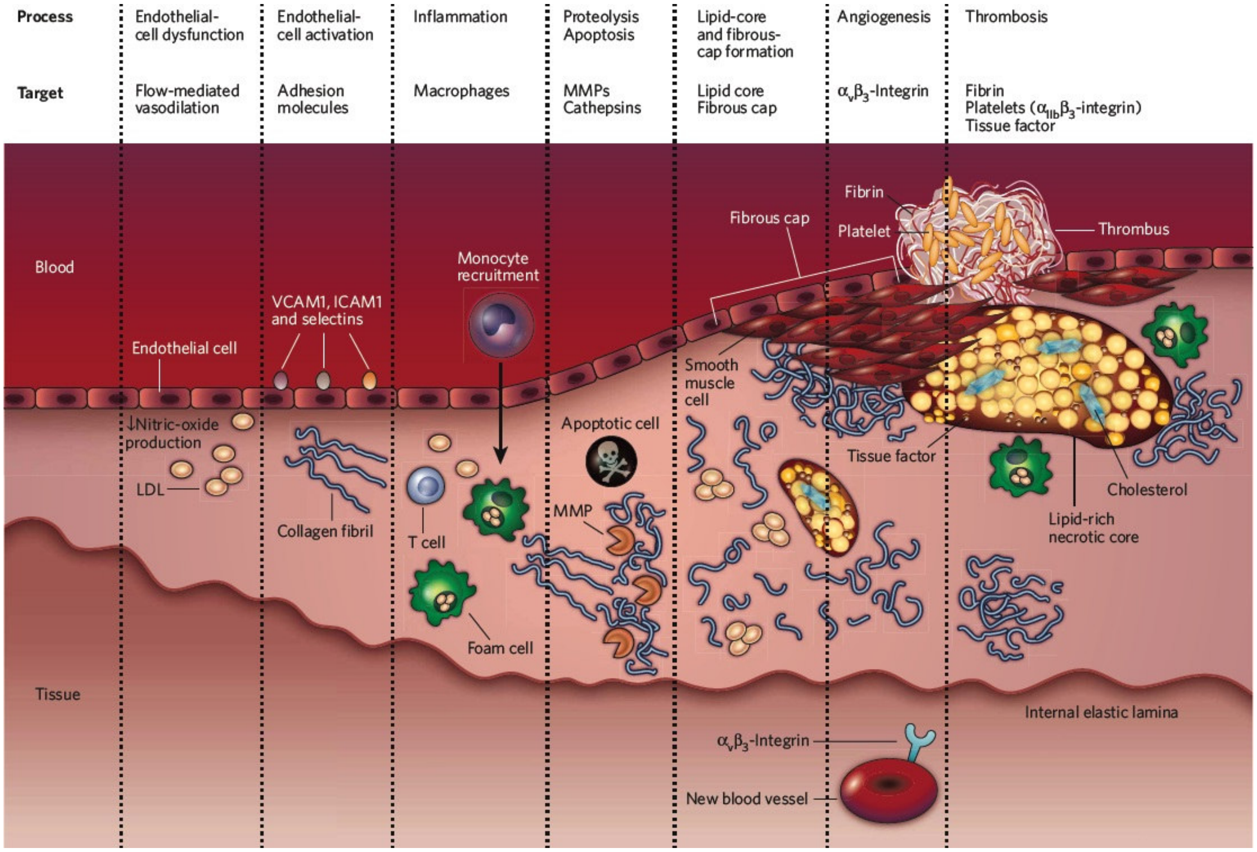

**FIGURE 2.** The development, progression, and subsequent thrombosis of atherosclerotic plaque[22]. With permission.

time[28,29]. However, it is noteworthy that a MION study carried out by Johansson et al. found the image enhancement to be markedly less effective than that observed in the Botnar Gd study discussed earlier; the authors suggest that the inferior enhancement may be attributable to the IONs being limited to the clot surface[24,31].

A targeted ION approach has also been developed for the detection of renal inflammation by Serkova et al.[32]. The authors utilized lipid-encapsulated IONs functionalized with complement receptor type 2 (CR2), a recombinant protein containing the binding domain for C3d, itself a marker for acute inflammation. By comparing kidney relaxation time differences for targeted and untargeted IONs to lupus nephritis and wild-type mice, the authors could show that the functionalized nanoparticle accumulation was only occurring with CR2 probes in the inflammation model mice.

Another inflammation development for IONs was achieved by van Kasteren et al. who produced high-contrast glyconanoparticles consisting of carbohydrate constructed on a platform of cross-linked amine-functionalized iron oxide[33] (Fig. 3). These nanoparticles allow direct detection of endothelial markers E-/P-selectin (CD62E/CD62P) in acute inflammation. The authors applied the probes via intracerebral injections to selectin expressing activated endothelium and a 3-D gradient echo T2-weighted pulse sequence was used to observe the presence of the nanoprobes. This allowed observation of the accumulation of signal at the areas of induced inflammation.

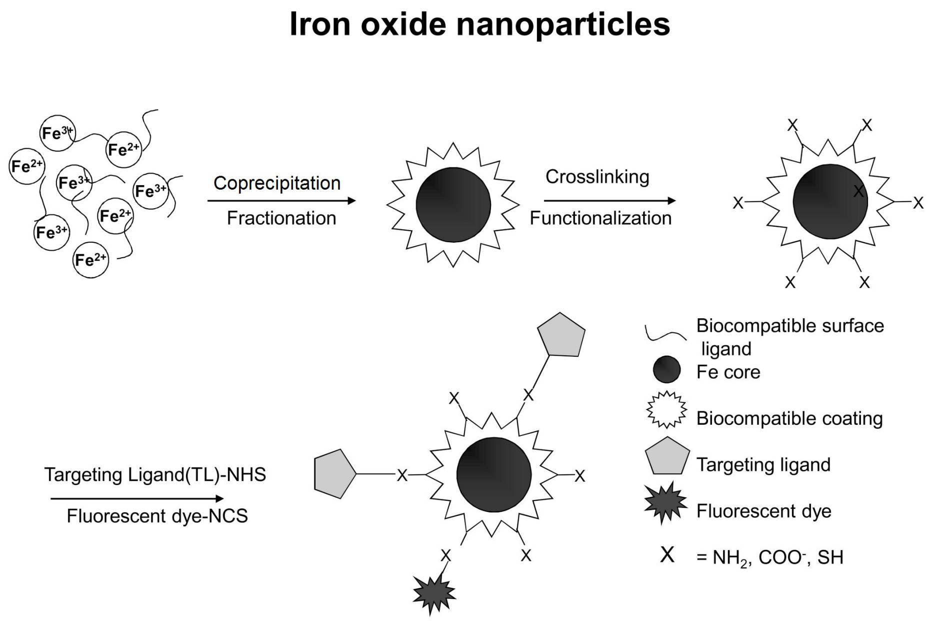

**FIGURE 3**. The synthesis (via coprecipitation) and functionalization of IONs.

## Fluorescent Approaches

Fluorescence detects and records the emission of light from a molecule that has been photonically excited by a source of different wavelength. It is a nondestructive technique, commonly applied to cells for tracking purposes. With respect to tissue, fluorescent imaging is predominantly used for *ex vivo* analysis or via invasive intravital microscopy. Fluorescence techniques are rarely applied to whole body scans due to inherent problems of working with tissue and light. A spectral region exists between 650 and 950 nm, known as the "clear window" for *in vivo* imaging[34]. At these longer wavelengths, autofluorescence and tissue damage is minimized, as is the adsorption of light from water and blood. Utilization of the window allows the most sensitive optical observation for fluorescence (and Raman, see later) signals from within the tissue. It should be noted that the longer wavelengths required are more readily scattered by tissue, making deep tissue imaging a particularly challenging technique.

Fluorophores can take many forms, but are generally proteins (e.g., green fluorescent protein, GFP), small molecules, or QDs. Researchers have also coupled the advantages of using fluorescence with magnetic nanoparticles, as discussed in the earlier section, by incorporating a fluorophore onto the surface of dextran-coated IONs. By using such an approach, probes were developed to study the manifestation of aberrant leukocyte infiltration of pancreatic islets, as observed with type 1 diabetes (T1D). With use of the magnetofluorescent nanoparticles, the authors developed a method to noninvasively measure the initiation and progression of insulitis in T1D mice models *in vivo* and in real time[35].

Sterically shielded liposomes (SSL) have been rendered fluorescently active by the introduction of a fluorophore below the PEG surface. Sandanaraj and coworkers exploited leaky vasculature in both tumor and arthritic mouse models with the use of SSL[36]. In the former approach, the authors injected samples i.v. into the tail, and carried out a temporal study to assess the accumulation of fluorophore at the specific site and throughout the whole animal. After 6 h, the fluorophore was distributed evenly throughout the whole organism. However, after 24 and at a peak of 48 h, the probe was shown to accumulate at the site of the tumor. When the analysis was carried to 72 and 96 h, the fluorescence signals had diminished and, significantly, no adverse toxic effects were observed with this study.

The most commonly applied fluorescent methods use QDs, small fluorescent nanoparticles that generally range in size from 2 to 10 nm. The optical and electronic properties of QDs are directly related to the particle size, thereby allowing control and tuning of these traits[37]. Comprised of a shell-encapsulated semiconductor core, QD cores are commonly combinations of semiconductors from groups II/VI (e.g., CdSe, CdS), IV-VI (e.g., PbSe, SnTe), and III-V (e.g., InP)[37]. The most commonly used shell materials are ZnS or ZnSe, allowing easy functionalization that can alter the QD properties, such as aqueous solubility and biocompatibility[37,38](Fig. 4). By utilizing a core material with a narrower bandgap than the shell, fluorescence activity is limited to the core region, thereby increasing the quantum yield of fluorescence and protecting the core material from photobleaching[37].

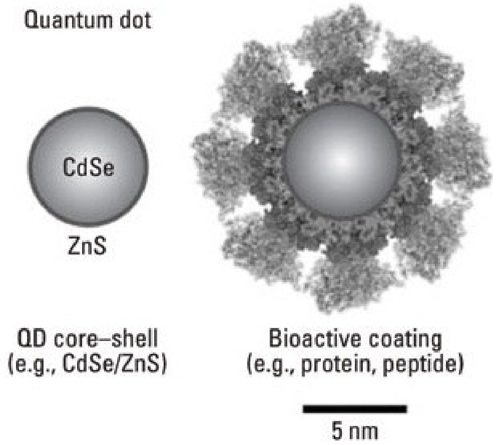

**FIGURE 4**. Illustration of a CdSe QD with an unfunctionalized ZnS shell and the same QD rendered biocompatible via surface functionalization.

Compared to conventional fluorescence techniques, QDs offer a number of significant advantages, including size-tunable properties, increased brightness, narrower emission profiles, and an easily customizable surface[37,38]. The most prominent drawback of QDs relates to the intrinsic toxicity of the core materials[39]. It is believed that QDs would likely be susceptible to highly oxidizing conditions *in vivo*, for example, during phagocytosis, and consequently this avenue of toxicity is of great concern[39]. It has been shown that the use of various surface coatings can reduce the cytotoxic nature of QDs, but their effects do not nullify it entirely[39]. The development of nontoxic QDs for biological application is a field of growing interest and a recent report by Pons et al. applied nontoxic NIR $CuInS_2$/ZnS to mice, allowing characterization of the immune response from the lymph node[40,41]. In comparison to Cd alternatives, the authors observed that a tenfold greater dose was required for the Cu QDs to illicit an immune response.

QDs are showing great promise in the fields of molecular imaging and medicine. With regard to the selective targeting and treatment of inflammation, QDs have been utilized successfully in a number of studies. Chakravarthy et al. coupled the drug doxorubicin (Dox) to CdSe/CdS/ZnS QDs and used them to target alveolar macrophages[42]. The authors observed that conjugation of Dox to the QDs offered numerous advantages in comparison to administering the drug as a free species. Uptake of Dox by alveolar macrophages was significantly enhanced by linking it to QDs. Further, the QD-Dox complex substantially reduced the observed inflammatory response and functional lung impairment when compared to the free drug. The latter result is especially significant as it suggests that QD-Dox had little effect on nonphagocytosing cells. The fluorescent functionality of the QDs was also used to image the administered QD-Dox via confocal microscopy, demonstrating that it retained its drug functionality after conjugation to the QDs. This study showed that QDs provide the basis for a novel, targeted, drug delivery system for the treatment of pulmonary diseases.

Costantini et al. utilized QDs with a view to developing a targeted treatment for inflammatory injury of the gut following a severe injury; in this case a severe, full-thickness burn[43]. Phage display was used to identify an amino acid chain that targets the injured intestinal epithelium (damage caused due to the inflammatory response initiated by the burn injury)[43]. This was then conjugated to the QDs, which allowed the fate of the targeting chain to be tracked again using confocal microscopy and led to visualization of the QDs accumulating in the gut mucosa following the burn injury. These results suggest that a targeted response could be developed for the treatment of gut injury following burns, trauma, and a number of other medical conditions, with greater efficacy than current treatments, and would be of great use in the medical world, as it is somewhat understood that gut injury acts as an initiator of SIRS (systemic inflammatory response) that can, in turn, cause distant organ injury and multiple organ failure[43]. It is noteworthy that these conjugated QDs have been administered into the lumen of the intestine, thus further work is needed to address if these are also active via other administrative routes. QDs have also recently been functionalized with eosinophil/mast cell chemokine receptor CCR3 for the detection of neovascular, age-related macular degeneration.

## SERS Probes

SERS is a sensitive optical technique characterized by molecularly specific spectra unique to the vibrational modes of a target analyte. Raman scattering requires the collection and observation of light inelastically scattered from a target molecule. It is an inherently weak process with an estimated 1 in every $10^6$ photons scattered in such a manner. The introduction of a strongly scattering, neighboring surface gives rise to SERS and reported sensitivity increases of greater than $10^{10}$ over the basic technique[44]. SERS sensitivity limits rival fluorescent alternatives and the sharp fingerprint spectra observed make the technique particularly amenable to multiplexed detection[45]. Unlike fluorescence, the spectra contain many molecularly specific sharp peaks, allowing the user to resolve multiple overlaid species (Fig. 5). Also, unlike fluorescence, SERS does not suffer from photobleaching, the loss of fluorescent signal that can be particularly challenging when working with multiple fluorophores. The sharp Raman fingerprint also has a major advantage when nanoparticles are applied to tissue. As discussed earlier, there exists a window between 650 and 950 nm where optical detection approaches in tissue are optimized due to minimal background interference. Fluorescent approaches have broad spectra that inhibit multiple species analysis in this window. With SERS, multiple species can be excited with the same wavelength, suggesting that for multiplexed detection in tissue, SERS provides a better platform.

SERS probes are most commonly prepared using gold or silver nanoparticles. Both are cheap to prepare, their size can be largely controlled during synthesis, and they provide an excellent scattering platform to observe SERS. Gold nanoparticles have been administered to combat inflammation in RA since the 1800s, and recent reports assessed and confirmed the therapeutic effects of the colloidal solutions[46]. The latest generation of gold (and silver) nanoparticles often uses a targeting molecule directly attached to the nanoparticle surface[47]. Although this approach can be used to generate a Raman fingerprint, most sensitive SERS signals are achieved with the introduction of a Raman tag also into the system. Raman tags are molecules that provide the best Raman response and are generally colored molecules containing multiple conjugated aromatic groups, commonly contained in fluorophores. This allows SERS researchers to borrow from much of the conjugation chemistry developed for fluorescence analysis, and the targeting molecule can either be conjugated to the Raman dye or mixed monolayers can be prepared directly on the nanoparticle surface. It is important that the fluorophore is as close as possible to the nanoparticle surface to quench fluorescence and prevent clouding of the Raman fingerprint.

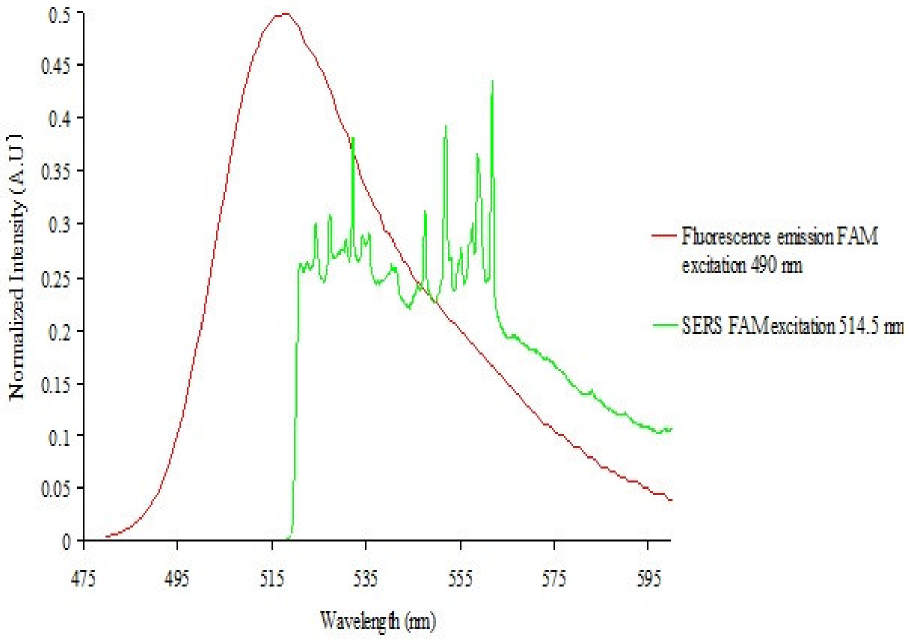

A

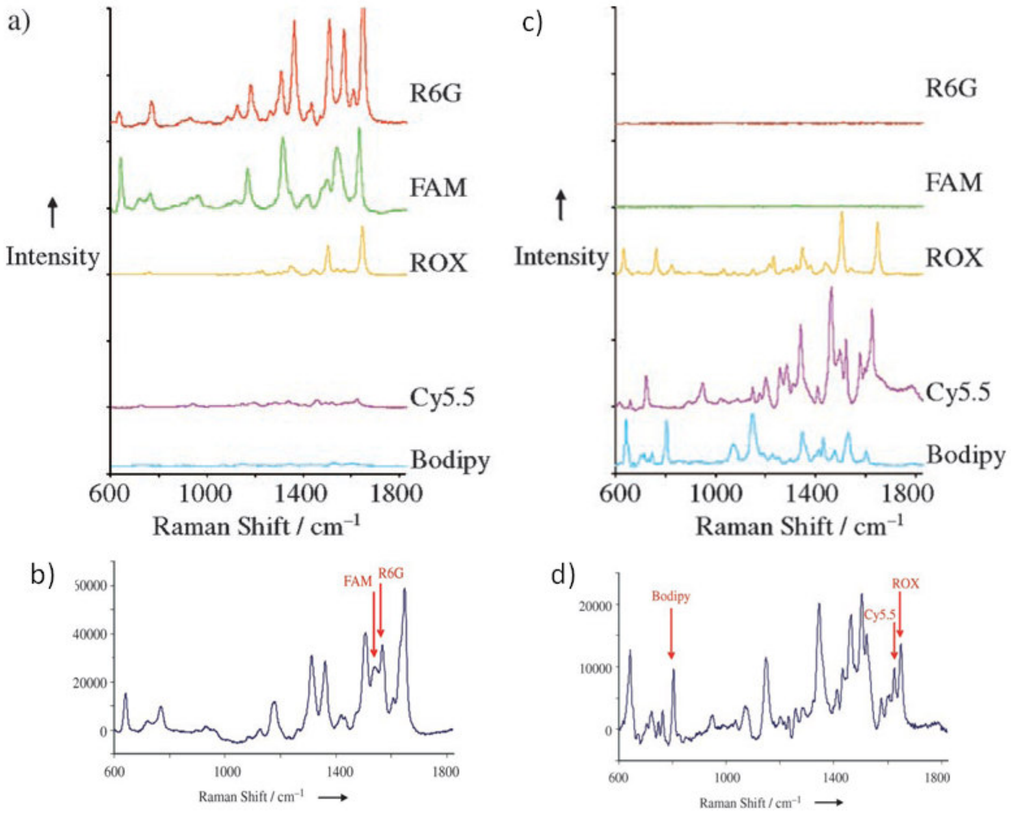

B

**FIGURE 5.** (A) The sharp vibrational bands observed with SERS in comparison to fluorescence. (B) The deconvolution of a multicomponent mixture using dual-wavelength SERS.

SERS nanoparticles have been applied to *in vivo* models for the detection of tumors expressing high levels of EGF receptors[48]. Briefly, EGF receptor antibody–functionalized gold nanoparticles were injected into tumor-induced mice. The authors found a localization of the probes in the spleen and liver; however, only the EGF receptor–targeted nanoparticles were located in the tumor as opposed to IgG control nanoparticles. To provide a glance for future multiplexing, Gambhir and coworkers have shown it possible to deconvolute the Raman signals from a four-nanoparticle mixture injected i.v. into a mouse model[49]. The samples were loaded into the tail vain as a complex mix, and their localization was confirmed in the spleen and liver of the animals, as shown in Fig. 6.

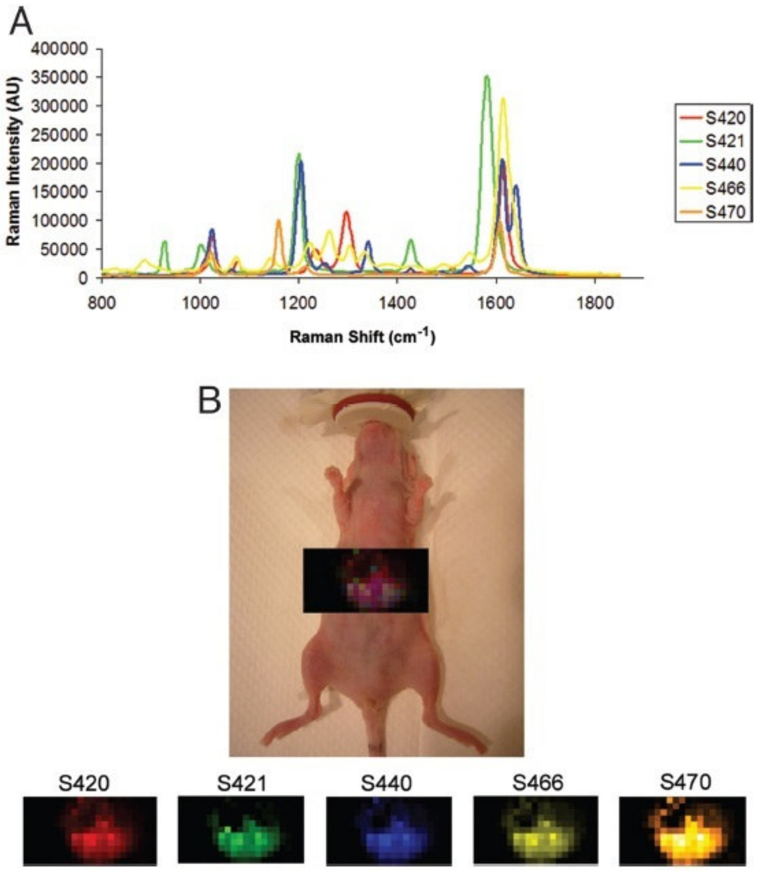

**FIGURE 6.** (A) The Raman spectra of five nanoparticle tags are overlaid to illustrate multiplexing potential. (B) False color representations of each Raman tag from (A) and its localization *in vivo*. The animal image shows all five colored tags overlaid.

## CONCLUSION

Chronic inflammation plays a pivotal role in the development of many diseases, including heart disease, Alzheimer's disease, arthritis, and some forms of cancer. Consequently, the ability to detect and treat such inflammation is critical in the treatment and prevention of associated afflictions.

Nanoparticle probes offer great promise in their application to image inflammation. Appropriate nanoparticle technologies can be utilized to detect and image areas of inflammation by acting as diagnostic probes. The ability to specifically target sites of inflammation may facilitate the detection of diseases in the preclinical stage; in turn, allowing earlier intervention and better-targeted treatment.

Furthermore, nanoparticles can be used to target and image selected biological structures and cells, such as macrophages, generating information with regard to the roles such cells play in the initiation and pathogenesis of inflammatory disease.

Nanoparticles have also been used as molecular vehicles for the delivery of pharmaceuticals and, in some cases, conjugation to a nanoparticle directly increases the efficacy of the payload. Exploitation of this facilitates the development of targeted, specifically tailored, treatments that are less damaging to nontarget areas.
